# Referential Values of Testicular Volume Measured by Ultrasonography in Normal Children and Adolescents: Z-Score Establishment

**DOI:** 10.3389/fped.2021.648711

**Published:** 2021-03-11

**Authors:** Chen Liu, Xiao Liu, Xiangxiang Zhang, Boyang Yang, Lan Huang, Hongying Wang, Hongkui Yu

**Affiliations:** ^1^Department of Ultrasonography, Guangzhou Women and Children's Medical Center, Guangzhou Medical University, Guangzhou, China; ^2^Department of Ultrasonography, Shenzhen Hospital of Guangzhou University of Chinese Medicine, Shenzhen, China

**Keywords:** testicular volume, Z-score, ultrasonography, children, referential values

## Abstract

**Objective:** To establish Z-score regression equation derived from age for testicular volume measured by ultrasonography in normal boys aged 0 to 18 years old.

**Method:** The length (L), width (W), and height (H) of 3,328 testicles from 1,664 Chinese boys were measured by ultrasonography. Lambert's formula: L × W × H × 0.71 was used to calculate testicular volume. Z-score regression equation derived from age was established by regression analysis of predicted values of testicular volume and standard deviations.

**Result:** There was no significant difference between left and right testicular volumes. Testicular volume was positively correlated with age, and logarithmic transformation of testicular volume can show a fine curve fit with age. To establish Z-score regression equation derived from age, the predicted values of testicular volume used cubic regression equations, and the standard deviation used square regression equations. The Z-score regression equation derived from age was calculated by the formula: z = [lg (L × W × H × 0.71) – (−0.3524-0.01759 × x+0.009417 × x2-0.0001840 × x3)]/(0.1059+0.01434 × x-0.0005324 × x2).

**Conclusion:** The current study provided a reference value for testicular volume of boys aged 0 to 18 years old. Z-score regression equation derived from age for testicular volume can be established. Z-score will be of great value for the testicular development assessment and disease diagnosis and follow-up.

## Introduction

Evaluation of testicular development is essential in male reproductive disorders, especially for children (1). Various common diseases, such as undescended testicles, varicocele, hydrocele, male pubertas praecox, etc., could compromise testicular volume, or even function (2, 3). In these diseases, monitoring testicular volume can provide valuable information for the assessment of disease progression, therapeutic efficacy, and might be helpful for choosing optimal surgery timing.

Although ultrasonography is a common method for testicular volume measurement, there is no ideal reference value currently (4). Unlike adults, the normal reference interval of testicular volume to children is not static. The development of normal reference interval for children should take age into consideration. In order to solve the above problem, many previous studies divided the age of children into intervals to calculate the normal reference range (5, 6). This method is not convenient and the results obtained are not accurate enough. Z-score regression equation and reference values have been widely used to quantify the departure from what is expected, also for the evaluation of physiologic or pathologic changes. Z-score based on ultrasonography measurements has gradually gained attention in various anatomic structures, especially in children under development phase. However, Z-score is less commonly utilized in the reproductive system, only a few studies have reported its application in uterus and ovary (7). To the authors' knowledge, there currently exists no age-based Z-score for testicular volume in pediatric population.

In this study, we aimed to determine comprehensive reference interval and Z-score regression equation for testicular volume in Chinese children from birth to 18 years of age. To do this, we used a standardized normalization protocol with thorough analysis of the normalized measurements for residual biases and adequacy Z-scores distribution.

## Materials and Methods

### Population

A total of 1,664 healthy children aged 0 to 18 were enrolled from Guangzhou Women and Children's Medical Center. There were several ways for recruitment: (1) children came for physical examination, (2) children admitted for diseases with no affection on testicles, (3) siblings or relatives of patients, (4) hospital staff's children. The inclusion criteria were as follows: (1) normal testicle and scrotum examined by ultrasonography, (2) clinical diagnosis without growth-related abnormalities, (3) testicle length, width and height obtained under standard ultrasonic procedure. The exclusion criteria were as follows: (1) children with any diseases have known to affect testicular development such as orchitis, testicular neoplasms, varicocele, cryptorchidism, hypogonadism, genetic disorders and testicular torsion; (2) unable to obtain standard measurements of testicle length, width and height. This study has been approved by the institutional review board of our institution. Written informed consent was obtained from a parent or guardian.

### Ultrasonic Image Acquisition and Data Processing

**Figure 1:**
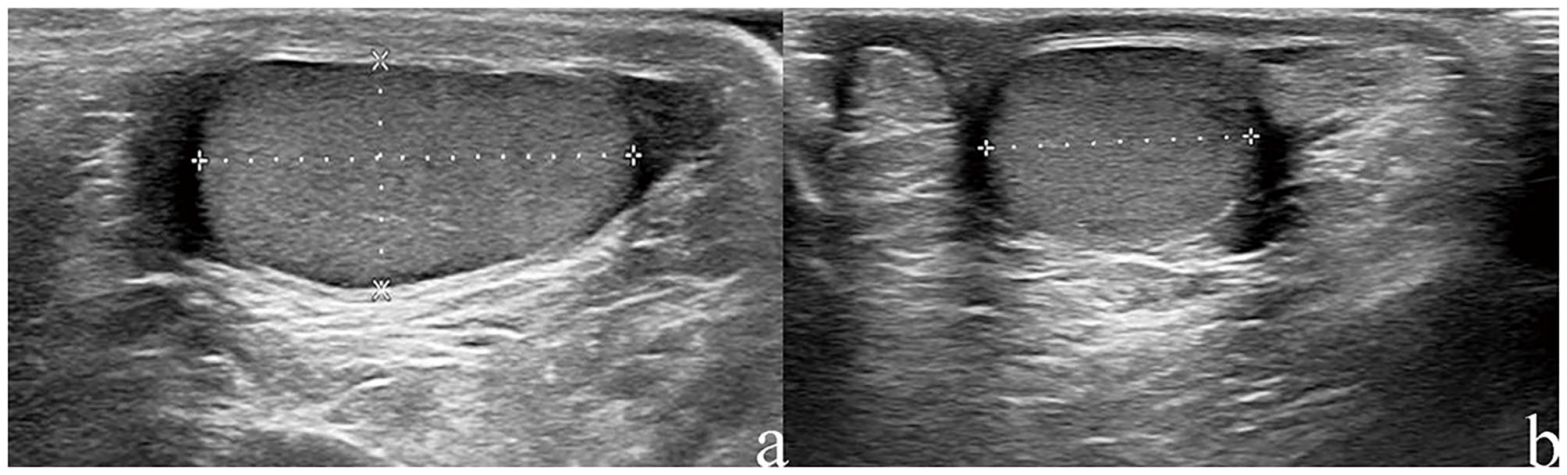
Measurement of testicle length, width, and height by Ultrasound on a 7-years old boy. The dotted lines show measurement of length and height in the longitudinal plane **(a)** and width in the transverse plain **(b)**.

Philip EPIQ7 ultrasonic machine equipped with a 7–12 MHz linear array probe was used for ultrasonography examination. The supine position was adopted during the examination. The uncooperative infants were sedated with chloral hydrate. The measurement of testicular volume was performed by four well-trained sonographers with more than 3 years of ultrasound work experience. The testicles were scanned gently with adequate gel to avoid distorting the testicular shape. The gray-scale images of the testicles were obtained in the longitudinal and transverse planes. The length (L) and height (H) in the longitudinal plane and width (W) in the transverse plane of the testicle were obtained (Figure 1). Each parameter was measured three times, and the highest values were adopted for calculation of testicular volume by Lambert's formula: L × W × H × 0.71.

### Intra-observer and Inter-observer Reproducibility Evaluation

Inter-observer and intra-observer agreement of the measurements were assessed in 50 randomly selected subjects. The same observer (C.L.) measured the testicular volume twice to assess intra-observer reproducibility. To assess inter-observer reproducibility, testicular volume measurements were performed by a second observer (HK. Y.), who was blinded to the results of the first observer (C. L.).

### Statistical Analysis

MedCalc (Version 18.2.1, MedCalc Software, Mariakerke, Belgium) was used for statistical analysis. Independent samples *t*-test were used to compare difference of testicular volumes between left and right sides. The function of age-related reference interval was to establish Z-score according to age. The method consists of the following steps: (1) If the distribution of the measurements (the variable for the establishment of a reference interval) presents skewness at different levels of age, the measurements are transformed logarithmically. (2) To establish the model on age from transformed measurements by using weighted polynomial regression (8). This regression model provides the predicted value of the (transformed) measurements as a function of age: y (age). (3) Calculated the residuals of this regression model. (4) The absolute residuals, multiplied by square root of π/2 are modeled on age by using weighted polynomial regression (9). This second regression model offers the standard deviation of the (transformed) measurements as a function of age: SD (age). (5) For each age within the observation range, the reference interval is calculated by taking mean (age) ± Z × SD (age). For a 95% reference interval z = 1.96. The resulting values are backtransformed to their original scale if the data were transformed in step 1. (6) The model is evaluated through analysis and plot of z-scores for all observations. The Z-score for an observed value y is calculated by z = [y – y (age)]/SD (age). The Z-scores should conform to a normal distribution. If they are not normal distributed, the model may not be adequate and other powers for the polynomial model may be selected. Normal distribution was evaluated with D'Agostino-Pearson test. Coefficient of skewness and coefficient of kurtosis are comprehensively used to analyze the normality of the Z-scores distribution. Two-tailed significance test was employed and *p* < 0.05 was considered statistically significant.

## Results

The left and right testicular volumes for each child were measured. The left testicular volume ranged from 0.18 to 19.17 ml, and the right testicular volume ranged from 0.16 to 19.78 ml. Left and right testicular volume in different age ranges are shown in Table 1. F-test showed that the left and right testicular volumes meet the homogeneity of variance (*P* = 0.605). Independent samples *t*-tests showed no statistically significant difference between left and right testicular volumes (*P* = 0.722). The statistical parameters of left and right testicular volumes and the result of independent samples *t*-test are shown in Table 2.

**Table 1:** The mean, standard deviation (SD), and 95% confidence interval of mean (95%CI) derived from testicular volume(ml) in different age ranges (year).

**Age group (y)**	***n***	**Left testicular volume (ml)**		**Right testicular volume (ml)**	
		**Mean ± SD**	**95% CI**	**Mean ± SD**	**95% CI**
0–1	93	0.43 ± 0.13	(0.41, 0.46)	0.44 ± 0.14	(0.41, 0.47)
1–2	158	0.45 ± 0.11	(0.43, 0.47)	0.45 ± 0.11	(0.43, 0.47)
2–3	128	0.53 ± 0.14	(0.51, 0.56)	0.54 ± 0.15	(0.52, 0.57)
3–4	110	0.57 ± 0.17	(0.53, 0.60)	0.57 ± 0.16	(0.54, 0.60)
4–5	109	0.63 ± 0.17	(0.60, 0.67)	0.64 ± 0.17	(0.61, 0.67)
5–6	108	0.63 ± 0.17	(0.60, 0.66)	0.65 ± 0.18	(0.61, 0.68)
6–7	104	0.71 ± 0.21	(0.67, 0.75)	0.72 ± 0.21	(0.68, 0.76)
7–8	92	0.78 ± 0.22	(0.74, 0.83)	0.77 ± 0.22	(0.73, 0.82)
8–9	110	0.88 ± 0.29	(0.82, 0.93)	0.90 ± 0.29	(0.84, 0.95)
9–10	127	1.25 ± 0.55	(1.16, 1.35)	1.26 ± 0.57	(1.16, 1.36)
10–11	138	2.48 ± 1.35	(2.25, 2.70)	2.57 ± 1.46	(2.32, 2.81)
11–12	110	3.24 ± 2.05	(2.85, 3.62)	3.37 ± 2.20	(2.96, 3.79)
12–13	84	5.01 ± 2.68	(4.43, 5.59)	5.13 ± 2.73	(4.54, 5.72)
13–14	51	6.39 ± 2.56	(5.67, 7.11)	6.71 ± 2.57	(5.98, 7.43)
14–15	41	7.74 ± 2.36	(7.00, 8.48)	7.83 ± 2.28	(7.11, 8.55)
15–16	36	9.72 ± 2.44	(8.90, 10.55)	9.73 ± 2.50	(8.88, 10.57)
16–17	34	11.53 ± 2.63	(10.61, 12.45)	11.44 ± 2.54	(10.55, 12.33)
17–18	31	13.41 ± 3.10	(12.28, 14.55)	13.53 ± 3.33	(12.30, 14.75)

**Table 2:** The statistical parameters of testicular volume (ml) and the result of *t*-test.

**Testicular volume**	***n***	**Lowest value**	**Highest value**	**Mean**	**Standard deviation**	**t**	***p***
Left	1,664	0.18	19.17	2.22	3.21	0.356	0.722
Right	1,664	0.16	19.78	2.26	3.25		

**Figure 2:**
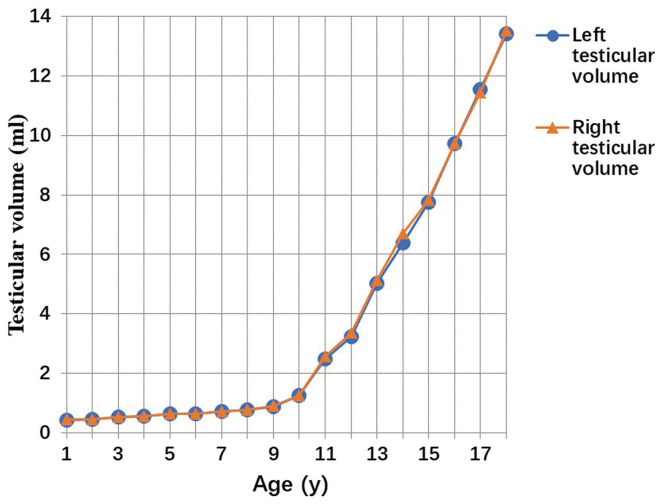
The line chart of left and right testicular volume.

**Figure 3:**
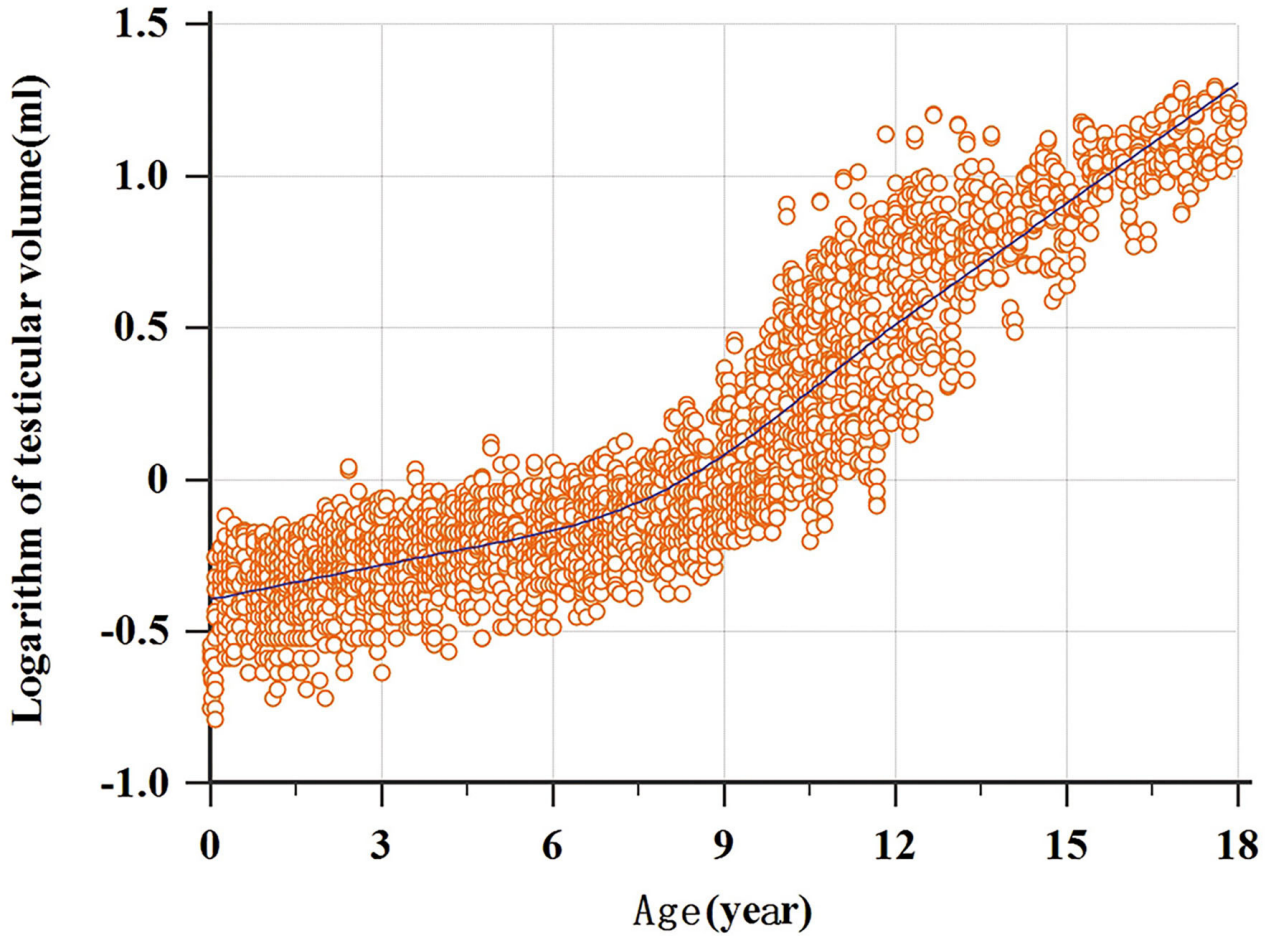
Scatter diagram by using logarithmic transformation of testicular volume.

**Figure 4:**
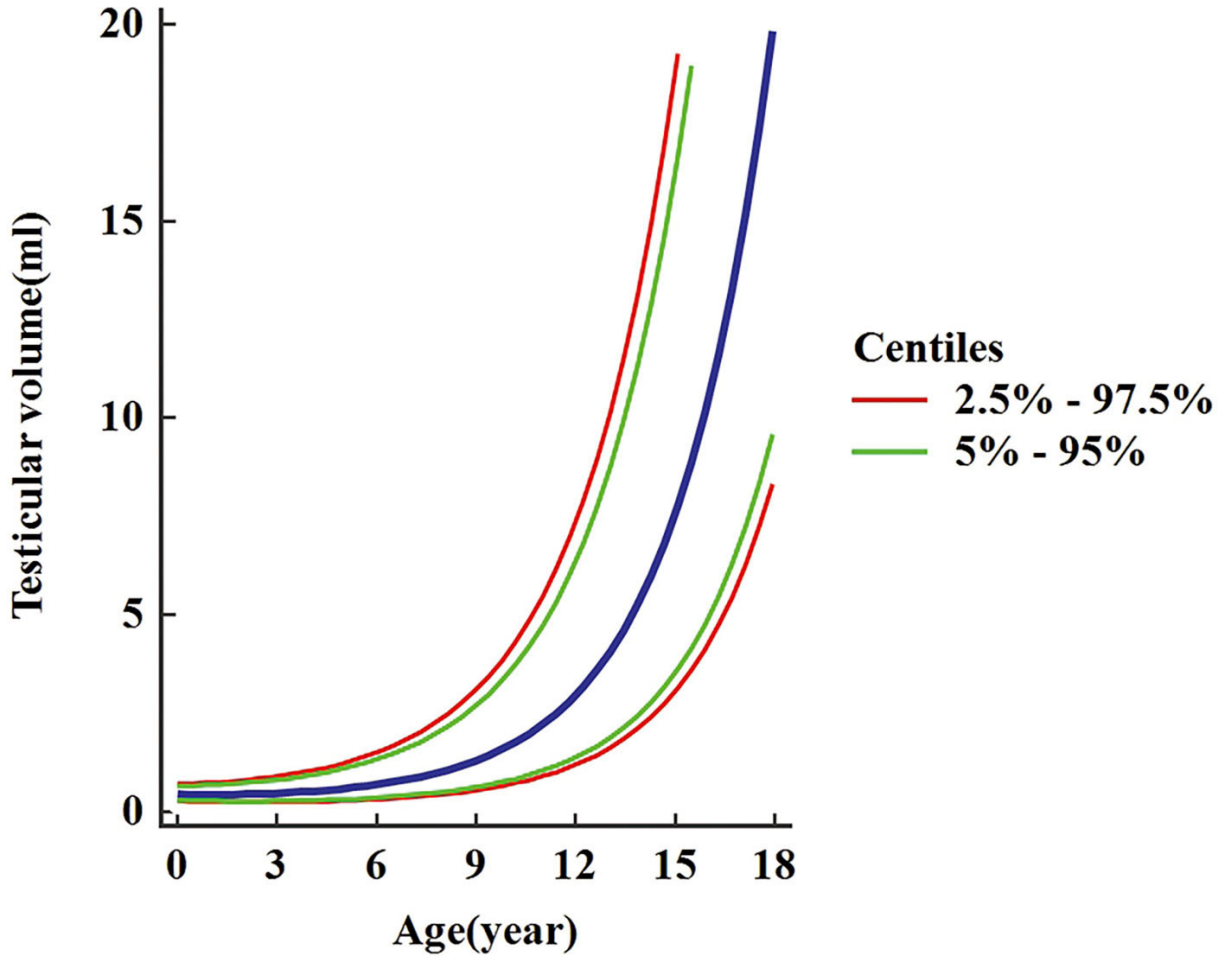
Regression results for the y (age) and percentile curve of testicular volume.

**Figure 5:**
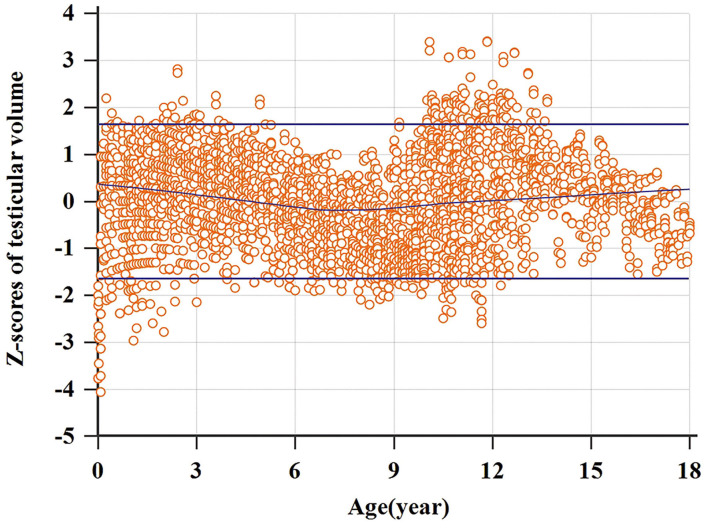
Diagram of Z score distribution.

A total of 3,328 testicular volumes (including the left and right testicular volumes) were measured to establish Z-score regression equation derived from age. The line chart of testicular volume was drawn by age (Figure 2). A base-10 logarithmic transformation was performed on testicular volume, and the scatter diagram shows testicular volume (transformed) as a function of age close to square or cubic curve (Figure 3). A cubic regression equation for y (age) and a square equation for SD (age) was established. The “x” represented age (in year). The parameters of regression equation and result of *t*-test are given in Table 3. Figure 4 presents regression results for the y (age) and fifth to 95th percentile intervals. The Z-score regression equation derived from age was calculated by z = [lg(L × W × H × 0.71)– (−0.3524-0.01759 × x+0.009417 × x^2^-0.0001840 × x^3^)]/(0.1059+0.01434 × x-0.0005324 × x^2^). The Z-scores were conformed to a normal distribution (*P* = 0.818). Figure 5 presents plots of Z-scores obtained by the above equation. Diagram of Z-score distribution displays 3,057 within the 90% interval and 271 outside the 90% interval. The coefficient of skewness and coefficient of kurtosis of the Z-score regression equation were (−0.01226, *P* = 0.7724) and (0.04513, *P* = 0.573), respectively.

**Table 3:** The correlated parameters of equation and result of *t*-test.

**Terms**	**Coefficient**	**t**	***p***
**Equation for y (age)**			
Constant	−0.3524		
x	−0.01759	−3.763	<0.001
x^2^	0.009417	13.644	<0.001
x^3^	−0.0001840	−6.681	<0.001
**Equation for SD (age)**			
Constant	0.1059		
x	0.01434	9.265	<0.001
x^2^	−0.0005324	−5.213	<0.001

### Intra-observer and Inter-observer Reproducibility

The equation used for the inter-observer variation was [(measurement observer 1-measurement observer 2)/mean of both measurements] ×100%, for the intra-observer variation both measurements were done by the same investigator.

The intra-observer variation for the testicular volume was 7% (left testicle: length 4%, width 6%, and height 5%, for right testicle: length 5%, width 7%, and height 9%) and the inter-observer variation was 9% (left testicle: length 7%, width 10%, and height 9% and right testicle: length 8%, width 9%, and height 9%).

## Discussion

In the current study, we established the latest normal reference value of testicular volume by analyzing ultrasonic parameters in 1,664 healthy children aged 0–18 years. On the basis of the large sample, we also developed the regression equation and Z-score calculation formula of testicular volume derived from age. To the best of our knowledge, this is the first study to apply Z-score as a normal reference interval for evaluating testicular development in Chinese children. This will offer convenience for physicians to assess the relative size of the testicles and their distribution in normal population.

Previously there existed few studies about normal reference value of pediatric testicular volume, with limitations such as small samples (10), inadequate age range (5) or performed decades ago (11). Moreover, none of these studies calculated Z-score to assess individual's value. Only Joustra et al. (12) developed reference charts for testicular volume and calculated SD score, but their subjects were mainly Europeans. There is no research on the normal reference value of testicular volume in Chinese children. The Z-score is a standard score that indicates by how many standard deviations a value is above or below the mean in a normal distribution. By definition, Z-scores must follow a standard normal distribution with a mean of 0 and a standard deviation of 1. Using the Z-score it is possible to say not only whether or not an individual result is statistically “normal,” but precisely how far that individual lies from the population mean. So far, the Z-score has been widely used in the development of children's echocardiographic index reference ranges, especially in research on Kawasaki disease (13–15), and on ultrasonography measurement of fetal intrauterine growth and development (16–18). However, the application of Z-score in reproductive system is rare. We accessed only one study in which Griffin et al. (7) established Z-score calculation formula and centile line for uterus and ovary. Our study developed the regression equation and Z-score calculation formula of testicular volume derived from age. In previous studies (14, 19), age, height, weight, and body surface area (BSA) can all be applied as independent variables of the Z-score regression equation. Although most researchers thought that the body surface area is better related to the development of human organs than age, height and weight (20), the measurement of height or weight and calculation formula of body surface area do have chances to bring bias. According to previous study (21), age is most closely related to testicular volume in healthy children, while other development indicators such as weight have no significant correlation with testicular volume. In the current study, we chose age as the independent variable of the Z-score regression equation.

In this study, we displayed left and right testicular volumes of different age groups. There was no significant difference between left and right testicular volume in children, which is consistent with previous studies (6). For the studies focusing on Z-score, larger sample brought more reliable results. Therefore, in this study, all testicular volume data were enrolled for analysis. No separate formula for the left or right testicular volume was established. Using the unified Z-score regression equation to evaluate testicular volume is more convenient in clinical applications.

In the Z-score regression equation, not only is it necessary to obtain estimation of testicular volume, the standard deviation is also an essential indicator. In previous study (14), a fixed standard deviation was hypothesized for convenience. This method did not take the heteroscedasticity of different age groups into account. Heteroscedasticity refers to the difference between the variances of dependent variables under different values of independent variables. Testicular volume development varied in different development phases, and the variance is also different. In this study, as with more studies on Z-score (15, 16, 19), the age-related standard deviation equation was adopted for calculation of Z-score.

Studies on male reproductive system (5, 22) have shown that testicular development needs to go through neonatal development period, resting period and maturation period. From birth to 5 months old, the testicles gradually increase in volume and double their weight and volume, and then develop very slowly or even shrink. This may be due to the disappear of gonadotropic hormones inhibition by the placenta at 3 to 4 months after birth, and then the secretion of gonadotropic hormones gradually decreases and tends to disappear. At ~9 years of age, the development of the reproductive system enters the adolescent development stage. The levels of related hormones rise rapidly, and the growth rate of testicular volume increases significantly. Research of Kuijper et al. (5) showed no significant change in children from 9 months to 6 years of age, which is similar with our study. We found only slow enlargement of testicles happened before 6 years old. No significant difference in testicular volume was observed in children aged 4 and 5. After 6 years old, the testicular volume increased rapidly, and grows even faster after 9 years of age. A previous study has shown (23) that testicular volume had a positive correlation with Serum inhibin B. Our study also confirmed that the testicular volume measured by ultrasonography could reflect the biological change of sex hormones, which in turn confirmed the accuracy of ultrasonography. Due to the regulation of testicular development, there is a significant inflection point on the curve of testicular volume with age. Establishing a simple linear regression formula directly cannot get a good fit, for the Z-scores does not conform to the normal distribution. In this study, the regression curve obtained by logarithmic transformation of the testicular volume can achieve a good fit, and the Z-scores obtained from the equations established here conform to the standard normal distribution.

The standard ultrasonic protocol of testicle and application of Lambert's formula were the reason why Z-score in this study had great value in clinical application. At present, testicular volume measurement by water displacement is considered the most accurate, while it cannot be used in clinical practice due to the requirement to remove the testicles (24). In clinical practice, the Prader Orchidometer is often used to measure testicular volume, but it will greatly overestimate testicular volume. Measuring testicular volume by ultrasonography is more accurate than traditional methods like Prader Orchidometer (25, 26). There are currently two commonly used formulas for calculation of testicular volume: (1) Ellipsoid formula: L × W × H × π/6; (2) Lambert's formula: L × W × H × 0.71. According to previous studies (24, 27, 28), the testicular volume calculated by Lambert's formula is very close to the actual testicular volume, while using the ellipsoid formula will significantly underestimate the testicular volume. Moreover, no matter what formula is used to calculate the testicular volume, there is a positive linear correlation with the actual testicular volume. Although the testicular volume estimated by the Lambert's formula may have some deviations from the actual testicular volume, it has no effect on the distribution of the Z-scores of the actual testicular volume.

Measurement of testicular volume is essential for assessing pubertal development and testicular function. Early or delayed development of puberty has an adversely impact on children. Children with delayed development of puberty always have psychological difficulties such as inferiority complex (29). Oehme et al. (25) concluded that the testicular volume of 2.7 ml measured by ultrasonography could be used as a marker of the beginning of puberty in boys, which was also consistent with the changes of sex hormone levels (30). A simple pubic hair staging could lead to a misclassification of puberty (31). Using the testicular volume to determine whether a boy starts puberty is the most reliable method in the beginning of puberty (32). Testicular volume is closely related to male reproductive system development and fertility prospects. For some children with benign tumors, testicular volume can gradually recover after partial orchiectomy. Testicular volume is also an important indicator for observing the process of testicular recovery after testicular preservation surgery (33).

In addition to assessing pubertal development, determining the testicular volume accurately is of great benefit for the evaluation of patients suffering from a variety of disorders impacting testicles. Testicular volume is considered to be of great importance in diseases such as undescended testicles (UDT), varicocele and hydrocele (2, 3). Studies have shown (34, 35) that the testicular volume of healthy children increases with age, and the situation differ in patients with UDT. In children with unilateral UDT, the affected testicular volume decreases, while the contralateral testicular volume may increase as compensation. When testicle is difficult to detect on one side, the increase in the volume of the contralateral testicle indicates severe atrophy or absence (36). Studies have shown (20) that testicular impairment resulting from varicocele has been significantly associated with lessened testicular volume. Testicular volume can be used to evaluate whether and when to treat varicocele. But for children with varicocele, there is currently no uniform cut-off value to determine when to perform surgery. A study (37) believes that a 20% difference in the volume of the two testicles can be an indication for surgical correction. Another study (38) suggestes that 15% should be used instead of 20% as the cut-off value. Although there was no significant difference between left and right testicular volume, the left testicular volume of most age groups in this study were smaller than the right testicular volume. This may be due to bilateral testicular asymmetry caused by very mild varicocele, which needs to be confirmed by further studies in future. A previous study has shown that (39) hydrocele can cause testicular edema, which leads to increased testicle. Ultrasonography is not only of great diagnostic value for hydrocele, but also is very helpful for the diagnosis of testicular edema. After the patient undergoes hydrocelectomy, the testicular volume will decrease and gradually return to normal. Testicular volume can also be monitored to observe the recovery of testicle (40).

There were several limitations in our study. First, the number of subjects over 16 years of age was relatively limited, which may have influence on the establishment of regression equation formula. Second, the selected children in this study did not routinely accept comprehensive examinations, we cannot completely exclude the disease that may have an impact on testicular growth. For example, we did not examine every subject in the upright position for physical examination with Valsalva maneuver during our research, so missed diagnosis of mild varicocele might not be totally avoided. Moreover, we did not assess the development of the correlation between testicular volumes and the hormonal, sexual maturity status, while other studies suggested testicular volumes may be also associated with Tanner stage.

## Conclusion

The current study provided a reference value for testicular volume of boys aged 0 to 18 years old. Z-score regression equation derived from age can be a promising solution for evaluation of testicular volume in children. It will provide valuable information for the assessment of testicular development, and also play an important role in disease diagnosis and follow-up.

## Data Availability Statement

The original contributions presented in the study are included in the article/supplementary material, further inquiries can be directed to the corresponding author/s.

## Ethics Statement

This study has been approved by the institutional review board of Guangzhou Women and Children's Medical Center. Written informed consent to participate in this study was provided by the participants'ylegal guardian/next of kin.

## Author Contributions

HY and CL: conception and design. HY, BY, XZ, and LH: acquisition of data. XL, HW, HY, and CL: analysis and interpretation of data. CL: manuscript drafting. XL, HW, and HY: manuscript revising. All authors contributed to the article and approved the submitted version.

## Conflict of Interest

The authors declare that the research was conducted in the absence of any commercial or financial relationships that could be construed as a potential conflict of interest.

## References

[1] Sotos JF Tokar NJ. Appraisal of testicular volumes: volumes matching ultrasound values referenced to stages of genital development. Int J Pediatr Endocrinol. (2017) 2017:7. 10.1186/s13633-017-0046-x 28725240 PMC5513322

[2] Sotos JF Tokar NJ. Testicular volumes revisited: a proposal for a simple clinical method that can closely match the volumes obtained by ultrasound and its clinical application. Int J Pediatr Endocrinol. (2012) 1:17. 10.1186/1687-9856-2012-17 22682237 PMC3538616

[3] Jedrzejewski G Wozniak MM Madej T Kryza R Zielonka-Lamparska E Wieczorek AP. The differences in testicular volumes in boys 8-36 months old with undescended, retractile and hydrocele testis–usefulness of scrotal screening ultrasound. Early Hum Dev. (2012) 88:185–9. 10.1016/j.earlhumdev.2011.07.023 21889272

[4] Goede J Hack WW Sijstermans K van der Voort-Doedens LM Van der Ploeg T Meij-de Vries A. Normative values for testicular volume measured by ultrasonography in a normal population from infancy to adolescence. Horm Res Paediatr. (2011) 76:56–64.10.1159/000326057 10.1159/000326057 21464560

[5] Kuijper EA van Kooten J Verbeke JI van Rooijen M Lambalk CB. Ultrasonographically measured testicular volumes in 0-to 6-year-old boys. Hum Reprod. (2008) 23:792–6. 10.1093/humrep/den021 18281246

[6] Srinivas R Thomas RJ Sebastian T Kurian JJ. Testicular volume in a cohort of prepubertal Indian children. J Indian Assoc Pediatr Surg. (2008) 24:192–6. 10.4103/jiaps.JIAPS_100_18 31258269 PMC6568151

[7] Griffin IJ Cole TJ Duncan KA Hollman AS Donaldson MD. Pelvic ultrasound measurements in normal girls. Acta Paediatr. (1995) 84:536–43. 10.1111/j.1651-2227.1995.tb13689.x 7633150

[8] Altman DG Chitty LS. Design and analysis of studies to derive charts of fetal size. Ultrasound Obstet Gynecol. (1993) 3:378–84. 10.1046/j.1469-0705.1993.03060378.x 12797237

[9] Altman DG. Construction of age-related reference centiles using absolute residuals. Stat Med. (1993) 12:917–24. 10.1002/sim.4780121003 8337548

[10] Kunde M Kunze C Surov A Ruschke K Spielmann RP. Sonographische Bestimmung der Hodenmaße im Alter von 0 bis 18 Jahren [Evaluation of testicular volume in 0-to 18-year-old boys by sonography]. Urologe A. (2015) 54:1772–8. 10.1007/s00120-015-3810-7 25989874

[11] Loke KY Lee BW Tan SH Lun KC Lee WK Lyen K. Normal standard of pubertal development in Singapore school children. J Singapore Paediatr Soc. (1991) 33:126–32. 1812328

[12] Joustra SD van der Plas EM Goede J Oostdijk W Delemarre-van de Waal HA Hack WW. New reference charts for testicular volume in Dutch children and adolescents allow the calculation of standard deviation scores. Acta Paediatr. (2015) 104:e271–8. 10.1111/apa.12972 25664405

[13] Dallaire F Dahdah N. New equations and a critical appraisal of coronary artery Z scores in healthy children. J Am Soc Echocardiogr. (2011) 24:60–74. 10.1016/j.echo.2010.10.004 21074965

[14] Zhang YQ Chen SB Huang GY Zhang HY Huang MR Wang SS. Coronary artery indexed diameter and z score regression equations in healthy Chinese Han children. J Clin Ultrasound. (2015) 43:39–46. 10.1002/jcu.22176 24975134

[15] Wu PF Li RZ Zhang R Zhang W Li X Zeng S. Detailed echocardiographic measurements of individual chamber in a Chinese cohort of hypoplastic left heart syndrome and comparison with normal fetuses via z-score modeling. Ultrasound Med Biol. (2020) 46:557–65. 10.1016/j.ultrasmedbio.2019.11.010 31859018

[16] Luewan S Srisupundit K Tongprasert F Traisrisilp K Jatavan P Tongsong T. Z Score reference ranges of fetal cardiac output from 12 to 40 weeks of pregnancy. J Ultrasound Med. (2020) 39:515–27. 10.1002/jum.15128 31512764

[17] Mao YK Zhao BW Zhou L Wang B Chen R Wang SS. Z-score reference ranges for pulsed-wave Doppler indices of the cardiac outflow tracts in normal fetuses. Int J Cardiovasc Imaging. (2019) 35:811–25. 10.1007/s10554-018-01517-1 30623353

[18] Lee W Riggs T Amula V Tsimis M Cutler N Bronsteen R. Fetal echocardiography: z-score reference ranges for a large patient population. Ultrasound Obstet Gynecol. (2010) 35:28–34. 10.1002/uog.7483 20014329

[19] Mannarino S Bulzomì P Codazzi AC Rispoli GA Tinelli C De Silvestri A. Inferior vena cava, abdominal aorta, and IVC-to-aorta ratio in healthy Caucasian children: ultrasound Z-scores according to BSA and age. J Cardiol. (2019) 74:388–93. 10.1016/j.jjcc.2019.02.021 30952562

[20] Costabile RA Skoog S Radowich M. Testicular volume assessment in the adolescent with a varicocele. J Urol. (1992) 147:1348–50. 10.1016/S0022-5347(17)37561-4 1569681

[21] Handelsman DJ Staraj S. Testicular size: the effects of aging, malnutrition, and illness. J Androl. (1985) 6:144–51. 10.1002/j.1939-4640.1985.tb00830.x 3997660

[22] Nistal M Paniagua R González-Peramato P Reyes-Múgica M. Perspectives in pediatric pathology, chapter 3. Testicular development from birth to puberty: systematic evaluation of the prepubertal testis. Pediatr Dev Pathol. (2015) 18:173–86. 10.2350/12-09-1255-PB.1 25075859

[23] Pierik FH Vreeburg JT Stijnen T De Jong FH Weber RF. Serum inhibin B as a marker of spermatogenesis. J Clin Endocrinol Metab. (1998) 83:3110–14. 10.1210/jcem.83.9.5121 9745412

[24] Sakamoto H Saito K Oohta M Inoue K Ogawa Y Yoshida H. Testicular volume measurement: comparison of ultrasonography, orchidometry, and water displacement. Urology. (2007) 69:152–7. 10.1016/j.urology.2006.09.012 17270639

[25] Oehme NHB Roelants M Bruserud IS Eide GE Bjerknes R Rosendahl K. Ultrasound-based measurements of testicular volume in 6- to 16-year-old boys - intra- and interobserver agreement and comparison with Prader orchidometry. Pediatr Radiol. (2018) 48:1771–8. 10.1007/s00247-018-4195-8 29980860

[26] Ogundoyin OO Atalabi OM. Comparison between testicular volumes as measured with prader orchidometer and ultrasonography in Healthy Nigerian Newborns. Afr J Paediatr Surg. (2018) 15:93–6. 10.4103/ajps.AJPS_32_17 31290471 PMC6615003

[27] Hsieh ML Huang ST Huang HC Chen Y Hsu YC. The reliability of ultrasonographic measurements for testicular volume assessment: comparison of three common formulas with true testicular volume. Asian J Androl. (2009) 11:261–5. 10.1038/aja.2008.48 19151736 PMC3735018

[28] Mbaeri TU Orakwe JC Nwofor AME Oranusi CK Mbonu OO. Ultrasound measurements of testicular volume: comparing the three common formulas with the true testicular volume determined by water displacement. Afr J Urol. (2013) 19:69–73. 10.1016/j.afju.2012.11.004

[29] Golub MS Collman GW Foster PM Kimmel CA Rajpert-De Meyts E Reiter EO. Public health implications of altered puberty timing. Pediatrics. (2008) 121(Suppl. 3):S218–30. 10.1542/peds.2007-1813G 18245514

[30] Madsen A Oehme NB Roelants M Bruserud IS Eide GE Viste K. Testicular ultrasound to stratify hormone references in a cross-sectional Norwegian study of male puberty. J Clin Endocrinol Metab. (2020) 105:dgz094. 10.1210/clinem/dgz094 31697832

[31] Biro FM Lucky AW Huster GA Morrison JA. Pubertal staging in boys [published correction appears in J Pedaitr 1995 Oct;127(4):674]. J Pediatr. (1995) 127:100–2. 10.1016/S0022-3476(95)70265-2 7608791

[32] Oehme NHB Roelants M Saervold Bruserud I Madsen A Eide GE Bjerknes R. Reference data for testicular volume measured with ultrasound and pubic hair in Norwegian boys are comparable with Northern European populations. Acta Paediatr. (2020) 109:1612–19. 10.1111/apa.15159 31899821

[33] Patel AS Coley BD Jayanthi VR. Ultrasonography underestimates the volume of normal parenchyma in benign testicular masses. J Urol. (2007) 178:1730–2. 10.1016/j.juro.2007.05.096 17707012

[34] Lunstra DD Wise TH Ford JJ. Sertoli cells in the boar testis: changes during development and compensatory hypertrophy after hemicastration at different ages. Biol Reprod. (2003) 68:140–50. 10.1095/biolreprod.102.006510 12493706

[35] Shin HJ Lee YS Yoon H Kim MJ Han SW Kim HY. Testicular volume and elasticity changes in young children with undescended testes. Med Ultrason. (2017) 19:380–5. 10.11152/mu-1093 29197914

[36] Diamond DA Paltiel HJ DiCanzio J Zurakowski D Bauer SB Atala A. Comparative assessment of pediatric testicular volume: orchidometer versus ultrasound. J Urol. (2000) 164:1111–14. 10.1097/00005392-200009020-00048 10958754

[37] Sansone A Fegatelli DA Pozza C Fattorini G Lauretta R Minnetti M. Effects of percutaneous varicocele repair on testicular volume: results from a 12-month follow-up. Asian J Androl. (2019) 21:408–12. 10.4103/aja.aja_102_18 30604693 PMC6628742

[38] Van Batavia JP Badalato G Fast A Glassberg KI. Adolescent varicocele-is the 20/38 harbinger a durable predictor of testicular asymmetry? J Urol. (2013) 189:1897–901. 10.1016/j.juro.2012.11.011 23154205

[39] Mihmanli I Kantarci F Kulaksizoglu H Gurses B Ogut G Unluer E. Testicular size and vascular resistance before and after hydrocelectomy. Am J Roentgenol. (2004) 183:1379–85. 10.2214/ajr.183.5.1831379 15505307

[40] Adaletli I Kurugoglu S Kantarci F Tireli GA Yilmaz MH Gulsen F. Testicular volume before and after hydrocelectomy in children. J Ultrasound Med. (2006) 25:1131–8. 10.7863/jum.2006.25.9.1131 16929013

